# Gut microbiome metabolites as key actors in atherosclerosis co-depression disease

**DOI:** 10.3389/fmicb.2022.988643

**Published:** 2022-11-10

**Authors:** Xing-Xing Liao, Xiao-Yun Wu, Yu-Long Zhou, Jia-Jun Li, You-Liang Wen, Jun-Jie Zhou

**Affiliations:** ^1^School of Rehabilitation Medicine, Gannan Medical University, Ganzhou, China; ^2^School of Basic Medicine, Gannan Medical University, Ganzhou, China; ^3^Key Laboratory of Prevention and Treatment of Cardiovascular and Cerebrovascular Diseases of Ministry of Education, Gannan Medical University, Ganzhou, China

**Keywords:** microbiome metabolites, atherosclerosis, depression, gut microbiome, comorbid

## Abstract

Cardiovascular diseases, mainly characterized by atherosclerosis (AS), and depression have a high comorbidity rate. However, previous studies have been conducted under a single disease, and there is a lack of studies in comorbid states to explore the commonalities in the pathogenesis of both diseases. Modern high-throughput technologies have made it clear that the gut microbiome can affect the development of the host’s own disorders and have shown that their metabolites are crucial to the pathophysiology of AS and depression. The aim of this review is to summarize the current important findings on the role of gut microbiome metabolites such as pathogen-associated molecular patterns, bile acids, tryptophan metabolites, short-chain fatty acids, and trimethylamine N -oxide in depression and AS disease, with the aim of identifying potential biological targets for the early diagnosis and treatment of AS co-depression disorders.

## Introduction

Cardiovascular disease, mainly characterized by atherosclerosis (AS), is the most common cause of death worldwide and is expected to be the top four causes of death worldwide by 2030 (WHO, 2008). There is growing evidence that depression, which is included among the top five causes of disability worldwide (Collaborators, G.D.a.I.I.a.P, 2017), is an independent risk factor for the occurrence of cardiac events (Zellweger et al., 2004; Hare et al., 2014; Vaccarino et al., 2020). Studies have shown that depression is associated with a higher risk ratio for cardiovascular mortality than hypercholesterolemia and obesity, and are intermediate between the “Big Five” classical cardiovascular risk factors (Ladwig et al., 2017) and is a common complication in patients with atherosclerosis cardiovascular disease (ASCVD; Levine et al., 2021). Individuals with depression are at substantially increased risk of cardiovascular disease and death (O'Connor, 2018; Levine et al., 2021), and in particular, are strongly associated with one of the most common cardiovascular diseases–ASCVD (Jee et al., 2019; Sun et al., 2019). In a multiracial study in the United States, depression was independently associated with atherosclerotic cardiovascular disease risk in all age groups (Dibato et al., 2021). In another large retrospective cohort analysis, it was found that patients with premature ASCVD had poor physical and mental health, with female patients more likely to report clinical depression and therefore require mental health interventions (Jain et al., 2022). Given that the two are so closely related clinically, we refer to this state as atherosclerosis co-depression disease.

Most of the current research is aimed at a single disease, and now is the dilemma facing the patient usually with a variety of diseases, because the interaction between the disease is complex, and the use of multiple drugs may lead to poor efficacy, in addition to the study of comorbidity is less, so we try to study the pathogenesis of common direction from both, identify common key targets for intervention. It is now believed that depression and atherosclerosis occur by similar mechanisms, including inflammation (Chrysohoou et al., 2018), hypothalamic–pituitary–adrenal axis dysregulation (Gu et al., 2012), endothelial dysfunction (van Dooren et al., 2016; Münzel and Daiber, 2020), and other major causes, while the development of modern high-throughput technologies has provided technical support for the study of the gut microbiome, and an increasing number of studies have revealed that gut microbiome is key factors mediating the development of the host’s diseases, including depression and AS (Jonsson and Bäckhed, 2017; Durack and Lynch, 2019). The gut microbiome can interact with the host by influencing metabolites, which are intermediate or final products of microbial metabolism, either directly from the bacteria themselves or the diet or the transformation of host-derived substrates. In this review, we will focus on the critical role played by gut microbiome-derived metabolites in the pathogenesis of depression and AS, which may provide valuable information for future diagnostic and therapeutic options for AS co-depression disorders.

### The change in the gut microbiome is closely related to the occurrence of depression and AS

Early studies first noted that microorganisms located in the gut have some connection to the host’s central nervous system, which is referred to as the brain-gut axis (Rieder et al., 2017), which expanded the horizon for uncovering the potential pathogenesis of psychiatric disorders. Studies have shown that germ-free mice exhibit an overall defect in microglia, i.e., altered cell ratios and an immature phenotype that leads to impaired innate immune responses (Erny et al., 2015), as well as deficits in social cognition and social perception (Sherwin et al., 2019), and Gareau tested germ-free (GF) mice cognitively using a new object recognition experiment and a T-maze experiment, which showed low rates of exploration and spontaneous exploration, without show signs of non-spatial or working memory (Gareau et al., 2011), and these results fully confirm the findings of Cryan et al. that gut flora can modulate the developmental and functional status of the brain (Cryan and Dinan, 2012), and is one of the important influencing factors in the occurrence of depression. In addition to exploring the relationship between gut microbiome and CNS disorders such as depression using GF animals, the relationship between gut microbiome and AS was also confirmed through it. As found by Stepankova: compared to ApoE^−/−^ mice raised under conventional conditions, GF ApoE^−/−^ mice consuming the same low-cholesterol standard diet instead developed atherosclerotic plaques, suggesting that gut microbiome can protect mice from atherosclerosis (Stepankova et al., 2010). Interestingly, Kiouptsi in another study confirmed by GF mice that gut microbiome lowered plasma cholesterol levels in Ldlr^−/−^ mice fed a normal diet, but not in Ldlr^−/−^ mice fed a high-fat diet. The reason for this was that the high-fat diet led to cholesterol spillage, which masked the bacterial effect. In addition, he found that gut microbiome increase low-grade inflammation in the vessel wall and can promote the development of atherosclerosis (Kiouptsi et al., 2019). This does not contradict the results of the previous ApoE^−/−^ model article, as the gut microbiome consist of pathogenic and protective bacteria and which specific members of the microbiota are not well studied in terms of promoting cholesterol excretion or plaque formation, which is an interesting direction of research.

### Relationship between the gut microbiome and depression

Several subsequent studies have found that the gut microbiome is closely associated with the development of depression, one of the common psychiatric disorders (Simpson et al., 2021). Cheng et al. used microbiome-associated gene set enrichment analysis to identify gut microbiome associated with psychiatric disorders and showed that major depressive disorder (MDD) was associated with genus *Desulfovibrio* (*p* = 0.003), order *Clostridiales* (*p* = 0.004), family *Lachnospiraceae* (*p* = 0.007) and genus *Bacteroides* (*p* = 0.007; Cheng et al., 2020). By analyzing stool samples from 46 depressed patients and 30 healthy controls, Jiang et al. showed that MDD patients had increased levels of *Enterobacteriaceae* and *Alistipes* and decreased levels of *Faecalibacterium*, where *Faecalibacterium* was negatively correlated with the severity of depressive symptoms (Jiang et al., 2015). On the other hand, Lai et al. used a more advanced shotgun metagenomic sequencing on stool specimens from 26 MDD patients and 29 healthy controls and showed a significant decrease in the abundance of *Bacteroidetes* and a significant increase in the abundance of *Actinobacteria* in MDD patients, where it is noteworthy that *Bifidobacterium* levels were increased in MDD patients (Lai et al., 2021). While *Bifidobacterium* is a commonly used probiotic, this certainly suggests that we should try to control for extraneous factors affecting heterogeneity (e.g., a sample size of participants, dietary habits, clinical medication, and their condition, sequencing methods, statistical methods, etc.) in conducting microbiome studies. In another study, by testing the feces of depressed patients, a decrease in the abundance and diversity of gut microbiome was found, followed by gavage of fecal flora from depressed patients to microbiota-deficient rats, which revealed that rats subjected to flora transplantation showed behaviors characteristic of depression as well as physiological features of altered tryptophan metabolism (Kelly et al., 2016). More cases are shown in Table 1. All of the above studies confirm that alterations in the gut microbiome are potentially important factors in the pathogenesis of depression.

**Table 1:** Summary of the relationship between depression and gut microbiome.

Subject	Interventions	Results	Reference
MDD patients	—	*Actinobacteria*↑, *Bacteroidetes*↓	Zheng et al. (2016)
Depressed patients	Probiotic treatment;	*Ruminococcus gauvreauii*↑, *Coprococcus 3*↑, beta-diversity↑	Reininghaus et al. (2020)
Human genotypes and fecal metagenomes	—	*Morganella*↑, *Klebsiella*↑associated with MDD	Qin et al. (2022)
MDD patients	—	*Bacteroides* is negatively associated with depression	Strandwitz et al. (2019)
MDD patients	—	*Bacteroides*↑, *Blautia*↓, *Eubacterium*↓	Yang et al. (2020)
Mice	chronic unpredictable mild stress(CUMS)	Lactobacillus↓, Akkermansia↑	Li et al. (2019)
Rats	Gavaging *Escherichia coli*	induced depression	Li et al. (2022a)
Macaca fascicularis	—	Veillonellaceae↑，Lachnospiraceae↑，Ruminococcaceae↓↑	Zheng et al. (2021)
Mice	Transplantation microbiota	alleviated depressive-like behaviors	Zhang et al. (2019)
Mice	Chronic restraint stress	Enterorhabdus, Parabacteroides and Kyn levels in the brain are negatively correlated	Deng et al. (2021)

### Relationship between the gut microbiome and AS

The interaction between the gut microbiome and the central system has provided ideas for research in other disease areas and has attracted many researchers to work to uncover the link between the gut microbiome and atherosclerosis. Several studies have shown significant alterations in the structure and composition of the gut microbiome in patients with AS-related diseases. A study conducted in Sweden involving 12 patients and 13 controls, using intestinal macrogenomics, confirmed that the genus *Collinsella* was enriched in patients with atherosclerosis, while *Roseburia* and *Eubacterium* were enriched in healthy controls (Karlsson et al., 2012). In another large clinical study, Jie performed a genome-wide association study of feces from 218 patients with atherosclerotic cardiovascular disease and 187 healthy individuals and showed that *Enterobacteriaceae* and *Streptococcus* spp. were enriched in the feces of patients with atherosclerotic cardiovascular disease (Jie et al., 2017). In addition, a multi-omics analysis based on 161 patients with Coronary artery disease (CAD) and 40 healthy individuals (sequence of the V3-V4 region of the 16S rRNA gene and metabolomics) showed that the composition of both gut microbiome and metabolites changed significantly with the severity of CAD. The abundance of bacterial co-abundance group17 (e.g., several Gram-negative bacteria such as *Veillonella*, *Haemophilus,* and *Klebsiella*) increased with the increasing severity of CAD. Another study confirmed that the metabolic modules of taurine and hypotaurine were negatively correlated with CAD severity, which could suggest that certain bacteria may affect atherosclerosis by modulating host metabolic pathways (e.g., taurine, sphingolipids, and ceramides) as well as benzene metabolism (Liu et al., 2019). For example, *Roseburia intestinalis*, which stands out for its potential role in the treatment of numerous human diseases, including AS, through the production of SCFAs, has gained recognition (Nie et al., 2021). As Kasahara found in his experiments, the abundance of *Roseburia intestinalis* in genetically diverse mouse populations was negatively correlated with the development of atherosclerotic lesions, for which the atheroprotective effect was mediated, at least in part, by the production of butyrate (Kasahara et al., 2018). Studies on the role of the gut microbiome in regulating cholesterol metabolism, a risk factor closely associated with AS, have also received wide attention. The results of Le Roy’s experiments showed that the gut microbiome strongly regulates plasma cholesterol levels, hepatic cholesterol synthesis, and enterohepatic circulation, and screened bacterial species or taxa involved in regulating cholesterol homeostasis as *Betaproteobacteria*, *Alistipes*, *Bacteroides*, and *Barnesiella* (Le Roy et al., 2019). Interestingly, the mechanism behind the cholesterol-lowering properties of Pu-erh tea lies in the fact that the Theabrownin in Pu-erh tea can act by inhibiting microbes associated with bile-salt hydrolase activity (Huang et al., 2019). In another animal experiment, the use of peptides reduced plasma total cholesterol levels and atherosclerotic plaque formation in Western diet-fed LDLr^−/−^ mice, and this therapeutic effect was eliminated when the gut microbiome was depleted by antibiotics (Chen et al., 2020a). More details can be found in the review published by Vourakis (Vourakis et al., 2021), which focuses on the current knowledge about the potential mechanisms by which microbial metabolites regulate cholesterol homeostasis, providing therapeutic strategies to reduce the risk of AS-related diseases. In addition, more cases can be found in Table 2. These results also confirm that certain alterations in the gut microbiome are potentially important factors driving the development of AS.

**Table 2:** Summary of the relationship between atherosclerosis and gut microbiome.

Subject	Interventions	Results	Reference
CAD patients	—	*Bacteroides vulgatus*↓, *Bacteroides dorei*↓	Yoshida et al. (2018)
Atherosclerotic patients	—	*Lachnoclostridium*↑, *Clostridium*↑	Cai et al. (2022)
CAD patients	—	associated with *Bacteroidetes*↓ and *Alistipes*↓	Choroszy et al. (2022)
Carotid atherosclerosis patients	—	most abundant species: *Bacteroides eggerthii, Escherichia coli*, and *Klebsiella pneumoniae*	Chen et al. (2021)
Atherosclerotic patients	—	*Bacteroides xylanisolvens*, *Odoribacter splanchnicus*, *Eubacterium eligens*, *Roseburia inulinivorans*, and *Roseburia intestinalis* decreased	Liu et al. (2020)
Mice	Transplantation microbiota	accelerates atherosclerosis	Brandsma et al. (2019)
Mice	—	Abundance of *Roseburia* sp. is inversely correlated with atherosclerotic lesion size	Kasahara et al. (2018)
Mice	synthesizing self-assembling cyclic D,L-α-peptides	inhibited AS	Chen et al. (2020a)
Mice	Antibiotics	increase the extent of atherosclerosis, associated with *Bacteroidetes* and *Clostridia*	Kappel et al. (2020)
Mice	Berberine	*Lachnospiraceae* NK4A136 group↑, *Bacteroidales* S24-7 group↑, *Eubacterium*↑, attenuates choline-induced atherosclerosis	Li et al. (2021)

### Study of the gut microbiome in depression co-cardiovascular disease

Recent articles have systematically elaborated on the common underlying pathogenesis of depression and AS-related diseases, including gut microbes (Wu et al., 2021). Similarly, the key role of gut microbes has also been demonstrated in several studies of depression in combination with cardiovascular disease. Kemp used an exposure to early life stress model to investigate the relationship between altered gut microbiome and depression and cardiovascular disease, showing that the model reduced microbial alpha diversity and altered microbial composition (Kemp et al., 2021). A recent study found that dysregulation of gut microbiome composition contributed to the development of depression-like behavior induced by chronic myocardial infarction, suggesting that exogenous modulation of gut microbiome composition may be a potentially important strategy for treating depression-like behavior caused by adverse cardiac events (Zhang et al., 2022). Our previous study similarly showed that mainly *Desulfovibrio* and *Akkermansia* were altered within the gut microbiome of the AS co-depression mouse model compared to the Control group of mice and confirmed that some lipid metabolites in the brain are strongly associated with some bacteria (Hu et al., 2022). In addition to testing whether gut microbiome are altered in the context of AS co-depression disease, sun et al. used *Bifidobacterium lactis* Probio-M8 adjuvant therapy improved the clinical efficacy of coronary artery disease treatment as well as alleviated depression and anxiety in patients through targeted modulation of Gut-Heart/-Brain Axes (Sun et al., 2022) and the systematic description of the neuro- and cardioprotective effects of probiotics in clinical trials by Ciernikova in a recently published review (Ciernikova et al., 2021) further confirm the scientific validity of this hypothesis.

**Figure 1:**
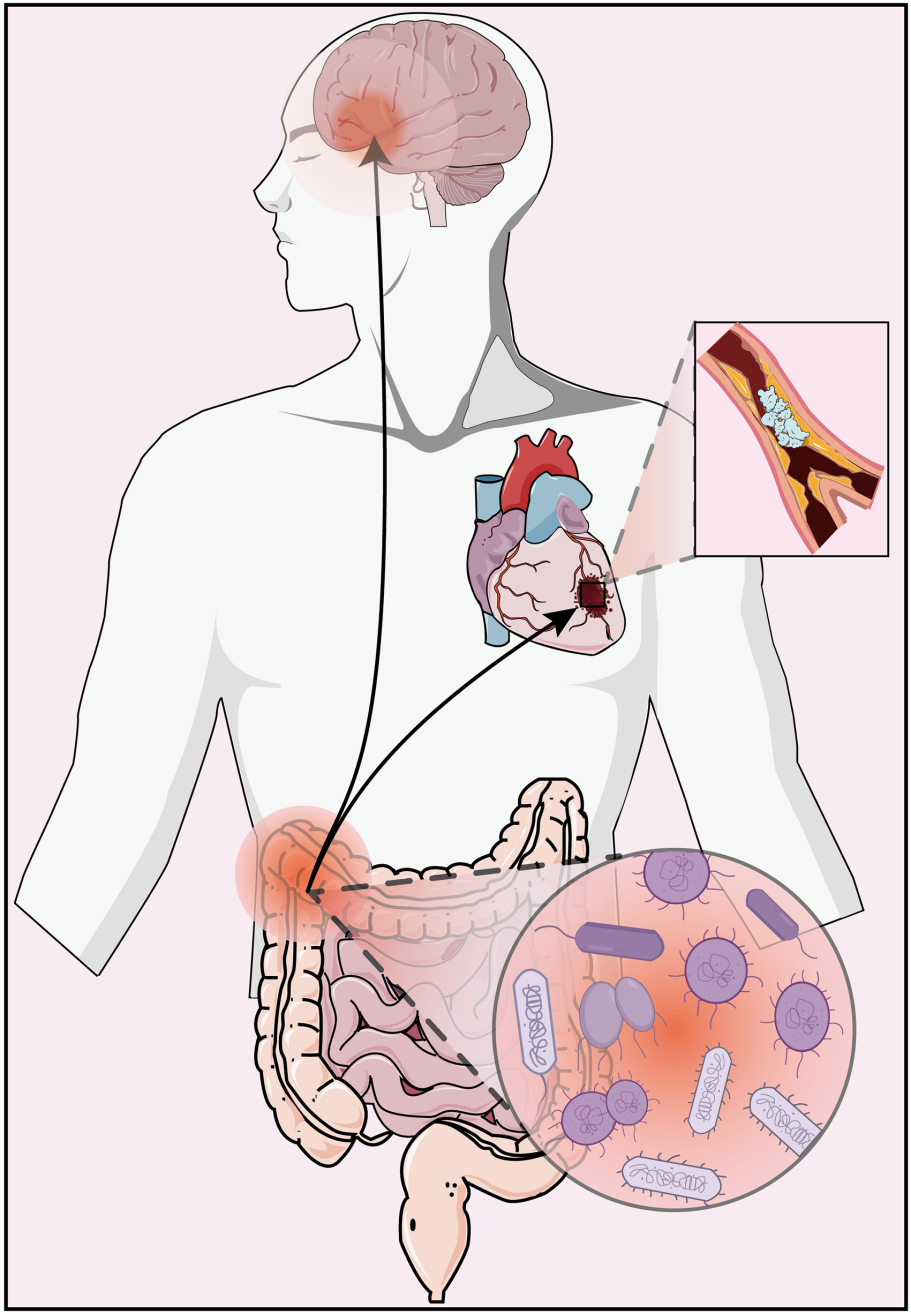
Dysbiosis of the gut microbiome can lead to depression and atherosclerosis.

These findings confirm that the gut microbiome plays a key regulatory role in the pathological development of depression and AS disease (Figure 1), both in pure disease and in comorbid states, and can provide new insights into the potential pathogenesis of AS co-depression and its therapeutic targets through regulation of gut microbiome.

**Figure 2:**
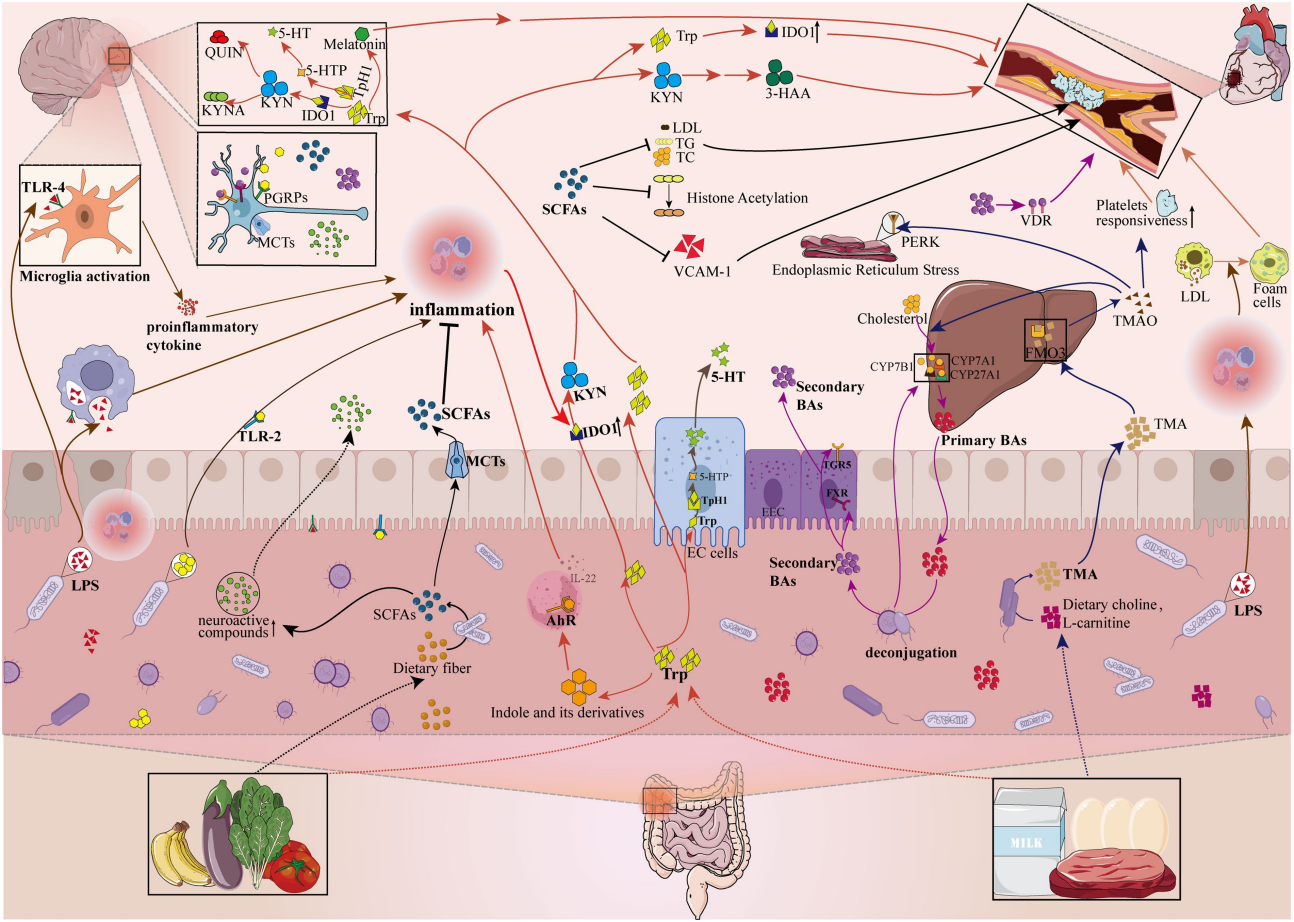
Gut microbiome disorders contribute to the pathologic development of depression and atherosclerosis by influencing metabolites. The gut microbiome can affect the pathological development of depression and atherosclerosis by participating in or mediating the production of metabolites (e.g., Pathogen-associated molecular patterns, Bile acids, Tryptophan metabolites, Short-chain fatty acids, Trimethylamine N-oxide). LPS, lipopolysaccharide; PGN, peptidoglycan; TLR-4, Toll-like receptors 4; TLR-2, Toll-like receptors 2; PGRPs, peptidoglycan recognition proteins; SCFAs, short-chain fatty acids; MCTs, monocarboxylic acid transport proteins; LDL, low-density lipoprotein cholesterol; TG, triglyceride; TC, total cholesterol; VCAM-1, vascular cell adhesion molecule-1; Trp, tryptophan; 5-HT, 5-hydroxytryptamine; KYN, kynurenine; AhR, aromatic hydrocarbon receptor; ECs, enterochromaffin cells; IDO1, indoleamine-2,3-dioxygenase 1; Tph1, tryptophan hydroxylase 1; 5-HTP, 5-hydroxytryptophan; KYNA, kynurenic acid; QUIN, quinolinic acid; 3-HAA, 3-hydroxyanthranilic acid; EEC, enteroendocrine cell; FXR, farnesoid X receptor; TGR5, Takeda G protein-coupled receptor 5; CYP7A1, cholesterol 7a-hydroxylase; CYP27A1, sterol-27-hydroxylase; CYP7B1, oxysterol 7α-hydroxylase; VDR, vitamin D receptor; TMA, trimethylamine; FMO3, flavin-containing monooxygenase isoform 3; TMAO, trimethylamine N-oxide; PERK, RNA-dependent protein kinase (PKR)-like ER kinase;

The available literature suggests that the interactions between the gut flora and the host are complex and not fully elucidated, but mainly include neural, the hypothalamic–pituitary–adrenal axis, immune and metabolic pathways (Grenham et al., 2011; Fung et al., 2017; Cryan et al., 2019). Next, we mainly elaborate on the important research results of several common and very important gut microbiome metabolites in the pathogenesis of AS and depression (Figure 2).

## Pathogen-associated molecular patterns

Pathogen-associated molecular patterns (PAMP) mainly refer to certain highly conserved molecular structures (e.g., lipopolysaccharide (LPS) and peptidoglycan (PGN)) on the surface of pathogenic microorganisms that induce host immune activity by binding to pattern recognition receptors (PRR) (Fitzgerald and Kagan, 2020). External causes such as a high-fat diet (Paone and Cani, 2020) and aging (Tran and Greenwood-Van Meerveld, 2013) can affect intestinal permeability directly or indirectly (causing gut microbiome disorders) and are important influences that contribute to PAMP leakage. Among them, LPS can bind to TLR4 in enterocytes to trigger the inflammatory process, further downregulating the level of tight junction proteins, resulting in more LPS translocation into the systemic circulation (Violi et al., 2022). LPS and PGN that enter the circulation can trigger a series of pro-inflammatory responses and are potential risk factors for inducing or/and promoting chronic inflammatory diseases such as depression and AS.

### The gut microbiome promotes depression through PAMP

Current studies have shown that LPS is one of the classical modeling modalities for conducting depressive disease studies due to its pro-inflammatory effects by participating in numerous signaling pathways closely related to depression, such as the autophagic pathway (Ali et al., 2020) or the activation of inflammatory vesicles (Arioz et al., 2019) or tryptophan metabolism (Walker et al., 2019) or Trkb/BDNF signaling (Li et al., 2022b). For example, systemic lipopolysaccharide administration induces the expression of IL-1β and other pro-inflammatory cytokine mRNAs and proteins in the brain, while IL-1β and TNF-α achieve 5-hydroxytryptamine uptake by stimulating synaptosomes in the midbrain and striatum of mice (Dantzer et al., 2008). Acute activation of TLR-4 (Toll-like receptors 4) by lipopolysaccharide or TLR-2 (Toll-like receptors 2) by peptidoglycan increases circulating levels of IFN-γ in mice, which can effectively activate indoleamine 2,3 dioxygenase (IDo) in the periphery and brain, resulting in a decrease in tryptophan levels (Lestage et al., 2002). And tryptophan levels are an important substrate for the synthesis of peripheral and central serotonin, which is one of the important guarantees for the normal functioning of the central nervous system and peripheral blood circulation system. On the other hand, peptidoglycan recognition proteins (PGRPs) are key sensing molecules in the innate immune system for the specific detection of bacterial peptidoglycan (PGN) and its derivatives and are considered potential key regulators of normal brain development and behavior. Bacterial peptidoglycans expressed on the cell walls of Gram-negative and Gram-positive bacteria can influence the development of social behavior by activating specific pathogen recognition receptors, such as PGLYRP2 expressed in the brain (Sherwin et al., 2019). Arentsen first discovered that peptidoglycan can affect the function of neutrophils derived from bone marrow, for which he experimentally found significantly lower levels of PGN in the cerebellum of GF male pups than in the SPF group, and detected the presence of PGN sensing molecules and PGN transporters in the brain, concluding that PGN can cross the blood–brain barrier under normal conditions (Arentsen et al., 2017). He next tested peptidoglycan recognition protein 2 (Pglyrp2) knockout (KO) mice to test the hypothesis that PGRPs play a role in motor control and anxiety-like behavior, and showed that both Pglyrp2 KO male and female mice exhibited anxiety-like behavior and that prefrontal cortex exhibited altered expression of genes related to synaptic plasticities, such as a significant increase in the expression of α-synaptic nuclear protein levels were significantly increased (Arentsen et al., 2018). It has been documented that it can inhibit tyrosine hydroxylase activity, which affects dopamine release (Somayaji et al., 2020), thus impeding normal brain functioning.

### The gut microbiome promotes as through PAMP

It has become a consensus that atherosclerosis is a chronic inflammatory disease and that LPS with pro-inflammatory effects enters the bloodstream through the compromised intestinal barrier, thus influencing known risk factors for atherosclerosis, such as platelet invasiveness, thrombosis, foam cell formation, inflammatory response, and oxidative stress (Chen et al., 2020b). A 10-year follow-up study of 2,452 patients found that high levels of lipopolysaccharide were significantly associated with coronary events with a risk ratio of 1.88 (1.13–3.12, *p* = 0.013, Q2-4 vs. Q1; Kallio et al., 2015). Low-grade endotoxemia is defined when the concentration of circulating levels of LPS is greater than 20 ng/ml. Endotoxin is involved in the thrombogenic process through several mechanisms, including upregulation of macrophage tissue factor expression (Violi et al., 2016) and amplification of platelet responses to common agonists in interaction with Toll-like receptors, stimulation of platelet secretion, and enhancement of platelet aggregation (Zhang et al., 2009; Tunjungputri et al., 2015), representing a novel pathway to amplify thrombus growth at the site of arterial lesions (Carnevale et al., 2020). For example, Jäckel et al. first experimentally demonstrated that the gut microbiome regulates hepatic von Willebrand factor (VWF) expression and plasma VWF levels through the PAMP-triggered TLR2 signaling pathway, thereby promoting VWF-integrin interactions on platelets and inducing arterial thrombus formation (Jäckel et al., 2017). In a subsequent in-depth study, they demonstrated for the first time that ADP-triggered activation of integrin α_IIb_β_3_ is regulated by commensal microbiota, and that integrin α_IIb_β_3_ synergizes with other platelet adhesion receptors and contributes to the deposition of type I collagen matrix under various conditions, playing an important role in thrombus formation (Kiouptsi et al., 2020). On the other hand, Zhou et al. conducted a clinical trial in 100 patients with ST-segment elevation myocardial infarction and confirmed that gut microbiome translocation leads to the accumulation of LPS in the circulatory system, and hypothesized that elevated LPS triggers monocyte recruitment, which activates systemic inflammation and ultimately leads to cardiac injury (Zhou et al., 2018). Similarly, Ramana also suggested that LPS-induced endotoxemia can produce myocardial depression (Ramana et al., 2006). In addition to confirming the association of LPS from gut microbiome with the development of AS, its pro-AS effect was similarly confirmed by direct injection of external LPS. In animal experiments, direct injection of LPS into rabbits accelerated the pathological process of cholesterol-induced atherosclerosis (Lehr et al., 2001), and by subcutaneously injecting mice with lipopolysaccharide for 1 month, it was shown that their fasting blood glucose, as well as the increase in systemic adipose tissue, were similar to those of high-fat-fed mice (Cani et al., 2007). Furthermore, as another common microbiome cell wall component peptidoglycan, its recognition is mediated by several families of pattern recognition molecules, including toll-like receptors, nucleotide-binding oligomerization domain-containing proteins, and PGRPs (Guan and Mariuzza, 2007). Peptidoglycan recognition protein-1 (PGLYRP-1) is part of the innate immune system that binds peptidoglycan and has attracted the attention of a wide range of researchers. Rohatgi measured PGLYRP-1 in 3222 subjects and reported for the first time that its circulating levels were associated with widespread subclinical atherosclerosis in humans; Among 2,443 patients without cardiovascular disease at baseline, elevated levels of circulating PGLYRP-1 at baseline were independently associated with an increased risk of a first ASCVD event (Rohatgi et al., 2009; Brownell et al., 2016), suggesting that the biological processes reflected by elevated PGLYRP-1 may be strongly associated with the development of clinical ASCVD.

## Bile acids

Bile acids, an important component of bile, are produced by the metabolism of host cholesterol in the liver and play an important role in fat digestion and energy metabolism. Circulating bile acids consist of primary bile acids produced by hepatic cholesterol and secondary bile acids formed by specific intestinal bacteria, which regulate their own and other substances’ metabolism by activating specific nuclear receptors (NRs) and G protein-coupled receptors (GPCRs), both as detergent molecules that facilitate nutrient absorption and as hormones that regulate nutrient metabolism (Ridlon et al., 2016). The biosynthesis of bile acids involves modification of the ring structure of cholesterol, oxidation and shortening of the side chain, and finally the coupling of bile acids to amino acids (Russell, 2003). The synthesis of bile acids relies on two main pathways: one is initiated by the cholesterol 7α-hydroxylation reaction catalyzed by the rate-limiting enzyme cholesterol 7a-hydroxylase (CYP7A1), which accounts for 75% of the total synthesis (Thomas et al., 2008); and the other is initiated by the catalysis of sterol-27-hydroxylase (CYP27A1; Russell, 2003), the formed 27-hydroxycholesterol is further hydroxylated by 7α-hydroxylase (CYP7B1; Thomas et al., 2008). Sayin et al. demonstrated that gut microbiome can regulate the expression of the enzymes cholesterol 7α-hydroxylase (CYP7A1), oxysterol 7α-hydroxylase (CYP7B1) and sterol-27-hydroxylase (CYP27A1; Sayin et al., 2013)，which subsequently mediate the synthesis of bile acids. Interestingly, gut microbiome are also extensively involved in bile acid conversion and metabolic pathways. One of the most studied microbially driven biotransformations is the bile salt hydrolase (BSH) activity of bacteria that undergo bile acid deconjugation. BSH is widely distributed in the major bacterial divisions and archaea species in the human gut, including *Clostridium*, *bifidobacterium*, *enterococcus*, *Lactobacillus*, *Bacteroides*, *Methanobacterium Smith*, *Methanococcus* and many other species, and is more abundant in the gut microbiome than in other microbial ecosystems. Besides deconjugation, intestinal microorganisms are the only source of 7α and 7β dehydroxylase activity, which produces “secondary” bile acids such as deoxycholic acid (DCA), lithic cholic acid (LCA), Hyodeoxycholic acid (HDCA) and ursodeoxycholic acid (UDCA; Wahlström et al., 2016). Overall, gut microbiome chemically diversify the bile acid pool through deconjugation, oxidation, exo-isomerization, 7α/7β dehydroxylation, esterification and desulfation, thereby allowing secondary bile acids to enter the portal circulation and function as endocrine-like signaling molecules with effective effects on host physiology and disease (Brown and Hazen, 2018).

### Bile acids are closely associated with depression

Conjugated and non-conjugated bile acids, as well as taurine or glycine, are potential neuroactive ligands (MahmoudianDehkordi et al., 2019; Spichak et al., 2021), such as ursodeoxycholic acid that can exert beneficial effects (MahmoudianDehkordi et al., 2019). The signaling of bile acids to the central nervous system includes direct and indirect pathways. The direct way is demonstrated by the fact that unconjugated and conjugated bile acids can cross the blood–brain barrier to reach the brain (Keene et al., 2001), where there are bile acid signaling mechanisms, i.e., receptors and transporter proteins capable of binding bile acids to transport them to neurons (Mertens et al., 2017). The indirect pathway is mainly triggered by the activation of the nuclear hormone receptor farnesoid X receptor (FXR) and Takeda G protein-coupled receptor 5 (TGR5). In line with this, both receptors, FXR (Huang et al., 2016; McMillin et al., 2016) and TGR5 (Keitel et al., 2010; Yanguas-Casás et al., 2017), in addition to being abundantly expressed in the enterohepatic circulation, have been detected in the brain. Interestingly, FXR knockout mice exhibited less depression-like and anxiety-related behaviors and altered neurotransmitter concentrations in different brain regions, such as an increased ratio of γ-aminobutyric acid to glutamate concentrations in the hippocampus, in addition to similar changes in serum and brain levels of various bile acids (Huang et al., 2015). Chen et al. similarly found that chronic unpredictable mild stress (CUMS) completely enhanced the expression of FXR protein and mRNA in the hippocampus, and overexpression of FXR in the hippocampus caused significant depression-like behavior and decreased expression of brain-derived neurotrophic factor (BDNF), while knockdown of FXR in the hippocampus completely inhibited the effects of CUMS on rat behavior and hippocampal BDNF expression (Chen et al., 2018). In addition, altered bile acid levels are closely associated with the development of central nervous system disorders. A study on Autism spectrum disorder found that reduced relative abundance of specific bacterial taxa (e.g., bile-metabolizing *Bifidobacterium* and *Blautia* species) was associated with deficient intestinal bile acid and tryptophan metabolism, significant gastrointestinal dysfunction, and social interaction impairment (Golubeva et al., 2017). By metabolomic analysis of the liver of depression model mice, Jia et al. found that initial bile acids play a key role in CUMS-induced depression in mice (Jia et al., 2016). In another study it was confirmed that abnormal activation of the secondary bile acid biosynthetic pathway thereby increasing the hydrophobicity of the bile acid pool, which in turn may contribute to the progression of metabolic disorders and depression-like behavior in CUMS mice (Qu et al., 2022). A recent review outlined the therapeutic potential of ursodeoxycholic acid and its conjugated species in neurological, neurodegenerative and neuropsychiatric disorders, affirming their positive anti-apoptotic, antioxidant and anti-inflammatory effects (Huang et al., 2022). These aforementioned findings provide new insights into the therapeutic options for depression.

### Bile acids play an important role in the pathogenesis of AS

In addition to acting on the central nervous system, there is evidence that bile acids act by binding to different receptors, including but not limited to facilitating lipid digestion, maintaining glucose, lipid and energy homeostasis, and inflammation (Witkowski et al., 2020; Brown et al., 2021). One host bile acid receptor that has received attention in recent years is the G protein-coupled receptor TGR5 (Watanabe et al., 2006; Pols et al., 2011), and TGR5 knockout mice are protected from the effects of atherosclerosis (Watanabe et al., 2006). Interestingly, certain bacterially modified bile acids (3-oxo-staphylococcal acid and staphylococcal acid) can also activate the vitamin D receptor (VDR), and genetic studies in both humans and mice have shown that VDR activation is associated with cardiovascular disease (Makishima et al., 2002). Notably, the hormone FGF19, which is secreted due to the activation of FXR by bile acid-binding, and its overexpression in the mouse brain leads to increased energy expenditure, and animals on a high-fat diet do not become diabetic or obese (Tomlinson et al., 2002), whereas the beneficial effect of systemic FGF19 on glucose metabolism is reduced by 50% when FGFR antagonists are injected into the brain (Morton et al., 2013), and the disruption of glucose metabolism is one of the factors contributing to the development of AS. In addition to binding to receptors, alterations in specific bile acid levels in cardiometabolic phenotypes and disease susceptibly have received a lot of attention. For example, alterations in plasma bile acid levels are associated with insulin resistance in type 2 diabetes, specifically referring to higher 12α-hydroxy/non-12α-hydroxy BA ratios were associated with lower insulin sensitivity and higher plasma triglyceride (Haeusler et al., 2013), in line with this, Gu et al. found that the treatment of diabetes was achieved by altering the relative abundance of microorganisms involved in bile acid metabolism, which in turn increased the ratio between primary and secondary bile acids and the level of unconjugated bile acids in plasma (Gu et al., 2017). Given the important regulatory role of bile acids in the development of AS, Xu et al. used activated transcription factor 3 to prevent atherosclerosis by inducing intrahepatic scavenger receptor group B type 1 and repressing cholesterol 12α-hydroxylase to interact with p53 and hepatocyte nuclear factor 4α, respectively, to regulate HDL and bile acid metabolism (Xu et al., 2021). Similarly, another study confirmed that by administering Resveratrol, the levels of genera *Lactobacillus* and *Bifidobacterium* could be increased, thus increasing the activity of bile salt hydrolases, which enhanced bile acid deconjugation and excretion to attenuate the formation of AS in mice (Chen et al., 2016). Overall, the above findings suggest that the gut microbiome can influence the development of AS co-depression pathology by affecting the synthesis and metabolism of bile acids.

## Tryptophan and derivatives

Tryptophan (Trp) is mainly ingested through food, and modern studies have found that the gut microbiome plays an important regulatory role in all three major pathways of Trp metabolism, including the 5-hydroxytryptamine (5-HT) pathway, the kynurenine (KYN) pathway, and the production of ligand-indole derivatives of the aromatic hydrocarbon receptor (AhR; Agus et al., 2018). However, it is not clear how the gut microbiome mediates the tryptophan metabolic pathway, but there is experimental evidence that microbiome metabolites such as secondary bile acids (Peregrin et al., 1999; Bunnett, 2014; Yano et al., 2015) and short-chain fatty acids (Fukumoto et al., 2003; Atarashi et al., 2013; Reigstad et al., 2015; Barbara et al., 2016) can act on Enterochromaffin cells (ECs) to promote the synthesis and release of serotonin. On the other hand, many microorganisms can synthesize serotonin directly from tryptophan (O'Mahony et al., 2015), which may be related to their ability to express tryptophan synthase. That gut microbiome is an important factor in 5-HT production is well documented in GF animal models. In one experiment, Sjogren found lower 5-HT concentrations in the blood of GF mice than in the normal group of mice (Sjögren et al., 2012), and another experiment similarly confirmed reduced 5-HT levels in the hippocampus of GF rats (Crumeyrolle-Arias et al., 2014). It is estimated that about 90% of Trp is used to produce KYN, which is mainly regulated by indoleamine-2,3-dioxygenase (IDO) or tryptophan 2,3-dioxygenase (TDO) in the KYN pathway (Gao et al., 2020). Furthermore, KYN and its metabolites, mainly kynurenic acid (KYNA) and quinolinic acid (QUIN), of which KYNA is considered to be a neuroprotective N-methyl-D-aspartic acid receptor (NMDA) receptor antagonist and QUIN is considered to be a neurotoxic NMDA receptor agonist (Pierozan et al., 2016), are closely associated with mental health (Cervenka et al., 2017). Some metabolites produced by intestinal microorganisms, such as SCFAs, particularly butyrate, are also known to regulate the KYN pathway (Kennedy et al., 2017), and Martin-Gallausiaux found that butyrate downregulates IDO-1 expression through a dual mechanism of reduced STAT1 levels and histone deacetylases (HDACs) inhibitor properties of SCFAs (Martin-Gallausiaux et al., 2018). In addition, Trp can be converted into several indole derivatives such as IAld and ILA by some *Lactobacilli* (Cervantes-Barragan et al., 2017). Many Gram-positive and Gram-negative bacteria encode a copy of the tryptophanase gene in their chromosomes and produce indoles, 85 species according to the count at that time (Lee and Lee, 2010).

### Studies of tryptophan and metabolites for depression

It has been shown that Trp can cross the blood–brain barrier and that circulating Trp from the periphery can affect Trp levels in the brain (Schwarcz et al., 2012). Messaoud found through clinical studies that low plasma Trp levels may be a biomarker of suicide in MDD and MDD patients and he suggested that reduced effectiveness of Trp for 5-HT synthesis and increased activation of the KYN pathway for associated with depression and suicide (Messaoud et al., 2019). As one of the tryptophan metabolites, 5-HT, is an important neurotransmitter involved in the control of adaptive responses in the central nervous system and associated with changes in mood, anxiety, or cognition (Canli and Lesch, 2007; Gonçalves et al., 2022), and plays an important role in neuronal differentiation and migration, as well as in axon growth, myelin and synapse formation (Gao et al., 2020), and the reduced availability of 5-HT in the brain is a depression a key feature of the pathogenesis of depression (Agus et al., 2018), consistent with the classic monoamine hypothesis in the etiological content of depression. One study found that male GF mice exhibited anxious behavior and significantly higher concentrations of 5-HT and its major metabolite 5-hydroxyindoleacetic acid in the hippocampus compared to conventional mice, suggesting that microbes can affect 5-HT neurotransmission in the central nervous system through humoral pathways (Clarke et al., 2013).

Many current studies confirm that depression is likewise a chronic inflammatory state. The Th1-type cytokine interferon-γ can lead to increased Trp catabolism and increased KYN/TRP ratio through activation of IDO activity (Yoshida et al., 1981; Taylor and Feng, 1991; Hwu et al., 2000). Increased levels of QUIN, one of the products of the KYN metabolic pathway, are closely associated with several distinguishing features of depression: reduced reaction time, cognitive deficits, and learning ability (Müller and Schwarz, 2007). QUIN can activate the NMDA receptor signaling pathway, leading to excitotoxicity and amplifying the inflammatory response, while KYNA is an antagonist of all ionotropic glutamate receptors and therefore could potentially block some of the effects of QUIN and other excitotoxins (Lim et al., 2017). In addition, KYNA is also a noncompetitive antagonist of low concentrations of alpha-7 nicotinic acetylcholine receptor (α7 nAChR), which is associated with learning and memory (Banerjee et al., 2012), and reduced KYNA levels may be involved in the pathophysiological mechanisms of depression by inhibiting α7 nAChR. When there is a relative imbalance between KYNA, which has neuroprotective effects, and QUIN, which has neurotoxic effects, it could explain the result that Meier et al. observed a reduction in medial prefrontal cortex thickness in MDD (Meier et al., 2016). Notably, Ogyu et al. showed reduced levels of KYNA and KYN in depressed patients by performing a meta-analysis of KYN pathway metabolite levels in depressed patients versus controls (Ogyu et al., 2018). The limitation of this study is that most of the reports included examined peripheral blood, but it is unclear to what extent peripheral KYN metabolites reflect the amount contained in the brain; after all, it is controversial whether each metabolite can cross the blood–brain barrier, or the amount that can enter the brain is inconsistent.

### Studies of tryptophan and metabolites in the development of AS pathology

5-HT was first identified from serum, also called serotonin. Very early studies have confirmed that platelets secrete serotonin, which promotes thrombogenesis, mitotic and proliferative processes in smooth muscle cells and is closely associated with AS development (Vikenes et al., 1999). Interestingly, Rami et al. found a pro-AS effect of SSRIs, confirming experimentally that SSRIs deplete major peripheral 5-HT stores mainly by inhibiting 5-HT reuptake transporter-mediated uptake in platelets (Rami et al., 2018), suggesting that peripheral serotonin levels are not the only key factor contributing to AS, which led us to focus more attention on related receptors. It has been noted that all 5-HT receptors, except the 5-HT_6_ type, are involved in cardiovascular regulation (Ramage and Villalón, 2008). Furthermore, it has been demonstrated that upregulation and/or increased sensitivity of peripheral 5-HT2A/1B receptors and downregulation of 5-HT transporter receptors may contribute to an increased risk of thromboembolic events in patients with depression and cardiovascular disease (Schins et al., 2003). Activation of the KYN pathway also plays an important role in atherogenesis. Song noted in his article that IDO activity in the blood positively correlates significantly with the progression of atherosclerosis (Song et al., 2017), and inhibiting IDO1 leads to more significant atherosclerotic lesions in ApoE^−/−^ mice fed a high-fat diet (Polyzos et al., 2015), while treatment of Ldlr^−/−^ mice with the tryptophan metabolite 3-hydroxycyanuric acid inhibits atherosclerosis by modulating lipid metabolism and inflammation (Zhang et al., 2012) is more evidence that activation of the KYN pathway can influence the pathological development of AS. On the other hand, a large fraction of Trp enters the indole pathway and is metabolized to Tryptamine and indole metabolites with signaling activity, which are subsequently involved in the pathogenesis of AS. For example, one of the products, indoxyl sulfate, is harmful to various cell types, including vascular endothelial cells (Hung et al., 2016), and has been shown to promote a procoagulant state *in vitro* and endothelial dysfunction *in vivo* (Dou et al., 2004) as well as aortic calcification (Adijiang et al., 2008), while indole-3-propionic acid and indole-3-aldehyde have anti-inflammatory protective effects (Paeslack et al., 2022). Direct injection of indole-3-propionic acid elevates blood pressure and increases cardiac contractility and cardiomyocyte metabolic activity (Konopelski et al., 2021). Indole derivatives act mainly through activation of AHR (Zelante et al., 2013), and AHR signaling is recognized to contribute to the development of AS-related diseases through inducing of IL-1β, IL-8 expression (Dahlem et al., 2020) and effects on different cell types closely related to atherogenesis (Wang et al., 2020), while treatment with AhR antagonists reduces the progression of atherosclerotic lesions (Wu et al., 2011) further confirms this important conclusion.

On the other hand, melatonin, located in the pineal gland, is similarly derived from the synthesis of L-Trp. There is growing evidence that melatonin has anti-inflammatory, antioxidant, hypotensive, and possibly anti-lipidemic properties (Dominguez-Rodriguez et al., 2010). It has been found that disorders of Trp metabolism resulting in melatonin deficiency may lead to abnormal hormone levels (e.g., aldosterone retention of water leading to increased blood pressure), which can lead to cardiovascular disease (Doi et al., 2010). In addition, melatonin has been shown to significantly improve antioxidant defense (increased catalase activity and reduced levels of thiobarbituric acid reactive substrates) and lipid-lowering (reduced LDL-C) and to lower blood pressure and inhibit plasma cholesterol levels in hypercholesterolemic rats (Koziróg et al., 2011). In line with this, Dominguez-Rodriguez et al. reported elevated levels of oxidized LDL and impaired nocturnal melatonin synthesis in patients with myocardial infarction, and this study was the first to confirm an independent correlation between oxidized LDL levels and melatonin levels at night in patients with myocardial infarction (Dominguez-Rodriguez et al., 2005). Overall, melatonin effectively interacts with various reactive oxygen species and reactive nitrogen species, and it also upregulates antioxidant enzymes and downregulates pro-oxidant enzymes, attenuating the molecular and cellular damage caused by free radicals involved in cardiac ischemia/reperfusion. These anti-inflammatory and antioxidant properties contribute to the prevention of atherosclerosis (Tengattini et al., 2008).

## Short-chain fatty acids

Short-chain fatty acids (SCFAs) are saturated fatty acids with carbon atoms ranging from one to six in length and are the main products of dietary fiber fermentation in the colon (Dalile et al., 2019). In the intestine, the conversion of dietary fiber to SCFAs involves a series of major reactions that are mediated by enzymes of specific members of the gut microbiome, and the end products are mainly acetic, propionic, and butyric acids (Koh et al., 2016). SCFAs are rapidly absorbed by colon cells mainly through active transport mediated by monocarboxylic acid transport proteins (MCTs), and the absorbed SCFAs pass through the blood circulation, reaching all parts of the body, including the brain (Dalile et al., 2019), which may be related to the high expression of MCTs on endothelial cells (Vijay and Morris, 2014). In addition, it has been found that propionic acid has a beneficial protective effect on the blood–brain barrier by inhibiting pathways associated with nonspecific microbial infections through a CD14-dependent mechanism and inhibiting LRP-1 expression to reducing harmful inflammation and oxidative stimulation (Hoyles et al., 2018), in line with this, treatment with SCFAs reversed the pathology of increased permeability of the blood–brain barrier in GF mice (Braniste et al., 2014).

### Studies related to SCFAs in depression

It has been found that butyric acid (Braniste et al., 2014) and acetate (Soliman and Rosenberger, 2011), inhibit the activity of HDACs and promote the hyperacetylation of histones, which are associated with neuropsychiatric disorders such as depression (Covington 3rd et al., 2009; Sarkar et al., 2014). Li et al. demonstrated by static and dynamic metabolomic analysis that propionic acid is a differential metabolite in CUMS rats, based on these findings, subsequent intrarectal administration of sodium propionate (the salt form of propionic acid) was used to confirm that propionic acid improved depression-like behavior in CUMS rats, which was linked to reduced catabolism of norepinephrine, tryptophan, and dopamine in the prefrontal cortex (Li et al., 2018b). In line with this, Marcel van de Wouw et al. found that short-chain fatty acids counteracted the lasting effects of chronic psychosocial stress and acted as antidepressants and anxiolytics by orally administering a mixture of three major short-chain fatty acids (acetic acid, propionic acid, and butyric acid) to mice (van de Wouw et al., 2018). The aforementioned studies have explored the direct therapeutic effects of SCFAs, and exploring the indirect therapeutic effects of SCFAs by focusing on the gut microbiome has also attracted widespread interest. For example, prebiotic administration increased cecum acetate and propionate concentrations and decreased isobutyrate concentrations, and these changes were significantly correlated with improvements in depressive behavior (Burokas et al., 2017). Another study found that treatment with electroacupuncture increased the relative abundance of SCFAs-producing bacteria, including *Ruminococcaceae*, *Phascolarctobacterium*, *Akkermansiaceae*, *Romboutsia*, and *Blautia*, which may be a result of electroacupuncture mediating SCFAs through the microbiota-gut-brain to improve evidence of anxiety and depression-like behaviors (Zhou et al., 2022). In addition to validation in animal experiments, the correlation between the concentration of SCFAs and the severity of depressive symptoms was even more revealed in a clinical trial, which found that the vast majority of SCFAs concentrations were higher in non-depressed women, with significantly lower levels of acetic acid, decreasing levels of propionic acid, and significantly higher concentrations of isocaproic acid compared to non-depressed women; Spearman correlation analysis showed that the concentrations of acetic acid and propionic acid were negatively correlated with BDI score (Skonieczna-Żydecka et al., 2018). As described by Oleskin, short-chain fatty acids act within intestinal endocrine cells, thereby stimulating the production of histamine, serotonin, 5-aminovaleric acid, and γ-aminobutyric acid, all of which are neuroactive compounds that are strongly associated with depressive-like behavior (Oleskin and Shenderov, 2016). Based on the analysis of the above findings, not all SCFAs have antidepressant effects, and there are contradictory results in some of the animal and human experiments; excluding the reasons for the differences between species, more experiments are still needed to investigate the specific substances that exert therapeutic effects.

### SCFAs as a potential strategy for the treatment of AS

Available evidence suggests that reliance on short-chain fatty acids produced by fermentation of dietary fiber by the gut microbiome may likewise be an effective preventive strategy for ameliorating atherosclerosis. Bartolomaeus et al. found a significant reduction in aortic atherosclerotic lesion area in propionic acid-treated ApoE^−/−^ mice (Bartolomaeus et al., 2019), and administration of *Lactobacillus* fermentum caused cecum microbiota alterations, increased colonic short-chain fatty acid levels, and reduced AS-related risk factors such as serum LDL, total cholesterol, and triglyceride levels (Yang et al., 2021). In addition to the hypolipidemic effect, Li et al. found that short-chain fatty acids could inhibit LPS or TNFα-induced endothelial inflammatory response and excessive vascular cell adhesion molecule-1 (VCAM-1) expression, which are two important steps in the development of atherosclerosis (Li et al., 2018d), and further studies found that SCFA could activate G-protein coupled receptor 41/43 and inhibit HDACs, playing a beneficial role in the treatment of AS-related diseases (Li et al., 2018c). Similarly, Shi et al. experimentally confirmed that the mechanism of Pae anti-AS is related to the improvement of Treg/Th17 balance in the spleen by increasing the production of microbiome-derived short-chain fatty acids (Shi et al., 2021).

## Trimethylamine N-oxide

In rodents and humans, gut microbiome enzymes convert choline and L-carnitine from food into a volatile gas called trimethylamine (TMA; Baker and Chaykin, 1962). TMA enters the liver through the portal circulation where it is converted to trimethylamine N-oxide (TMAO; Komaroff, 2018).

### TMAO is a strong predictor of AS-related diseases

TMAO production from phosphatidylcholine in feed has long been found to be associated with an increased risk of major adverse cardiovascular events, which is dependent on the metabolism of the gut microbiome (Wang et al., 2011; Koeth et al., 2013; Tang et al., 2013). Using non-targeted metabolomics as a platform for discovery, we further believe that TMAO is a strong predictor of AS-related disease by showing a causal relationship between TMAO and atherosclerosis (Wang et al., 2011). Interestingly, Lindskog Jonsson et al. later found experimentally that TMAO concentrations were not associated with atherosclerotic lesion size, and he attributed the contradictory findings of his experiments with those of previous investigators to a different experimental setup probably: previous studies used antibiotics to deplete the gut microbiome and started choline supplementation at weaning, whereas his experiments supplemented choline at 8 weeks of age when the atherosclerotic disease had already begun to develop. In addition, he suggested the interesting conjecture that dietary choline may be an important factor influencing the development of early atherosclerosis (Lindskog Jonsson et al., 2018). The mechanisms by which TMAO is thought to increase the risk of cardiovascular disease are diverse and include altered tissue sterol metabolism, enhanced endothelial cell activation and vascular inflammation, and pro-fibrotic signaling pathway stimulation (Roberts et al., 2018). Notably, the gut microbiome can directly promote platelet hyperresponsiveness and enhance thrombogenic potential through the production of TMAO (Zhu et al., 2016), and in subsequent human feeding studies, TMAO levels were significantly increased by 10-fold in healthy volunteers following oral choline administration, platelet reactivity and aggregation were enhanced, and a significant dose-dependent association was observed between plasma TMAO levels and platelet function (Zhu et al., 2017). These results suggest the possibility of using the TMAO pathway of the gut microbiome as a therapeutic strategy, such as inhibiting atherosclerotic plaque formation through dietary control, improving the microbial community with probiotics, or inhibiting key enzymes that produce TMAO and-moderating the platelet hyperreactivity associated with elevated TMAO, is worthy of further investigation.

### Advances in the study of TMAO in depression

TMAO promotes brain aging and cognitive impairment in addition to being an influential factor in cardiovascular disease (Li et al., 2018a). One study found that participants with PTSD symptoms had significantly higher TMAO levels immediately after acute myocardial infarction than patients without acute myocardial infarction symptoms and that TMAO could be a significant predictor of PTSD symptoms (Baranyi et al., 2021). In addition, a clinical trial based on 251 individuals found a positive correlation between serum TMAO levels and the severity of depressive symptoms (Meinitzer et al., 2020). Consistent with this, TMAO levels were significantly higher in depressed patients than in healthy controls (Liu et al., 2015). Although the above study only found peripheral TMAO levels to be associated with depressive symptoms, this suggests to us that TMAO is capable of contributing in some way to the development of central disorders. Coincidentally, Chen et al. experimentally identified the endoplasmic reticulum stress kinase PERK [RNA-dependent protein kinase (PKR)-like ER kinase] as a receptor for TMAO, which induces the endoplasmic reticulum stress signaling pathway by binding to the endoplasmic reticulum stress protein PERK (Chen et al., 2019), More importantly, endoplasmic reticulum stress has been shown in many studies to mediate one of the pathogenic mechanisms of psychiatric disorders such as depression (Gold et al., 2013; Xiang et al., 2017). In addition to the detection of TMAO levels alone, Zheng et al. similarly demonstrated that depression patients had altered concentrations of gut microbiome metabolites, including TMAO (Zheng et al., 2013). Overall, these studies support the association of TMAO with the development of depression and provide new treatment options for the treatment of depression.

## Conclusion

The time has come to study microbiome metabolites, mainly PAMP, bile acid, tryptophan and derivatives, SCFAs, and TMAO, which play a central role in the pathophysiology of depression and AS. There is reason to believe that this is potential pathogenesis common to both diseases, depression and AS, and that some of these factors may serve as potential biological targets for early diagnosis and treatment of AS co-depression disorders. We can expect that soon, patients with AS co-depression can get rid of the physical and mental burden caused by multiple drug use, which is an important and valuable research direction that needs to be explored deeply.

## Limitations

In this review, due to the limited existing research results related to AS co-depression, we only elaborated on the important research results of each microbiome metabolite in depression and AS disease, respectively. Due to the interaction between two kinds of the disease being complex and has not yet been studied clearly, the studies in a separate state of disease are flawed, and more researchers are required to conduct more in-depth follow-up experiments in the state of AS co-depression. In addition, the gut microbiome can communicate with its hosts in both directions and can influence each other, and the ways of communication include neurological, immunological, and metabolic pathways. This review simply describes how gut microbes affect the host’s disease by mediating metabolites in a unidirectional manner, and only a few common metabolites are described to provide the reader with a preliminary overview of the critical role of gut microbiome metabolites in AS co-depression diseases and to provide ideas for researchers interested in this field. Finally, it is still unclear which specific bacteria and the metabolites mediated by them are most conducive to improving the state of AS co-depression, which needs to be further verified in larger preclinical and clinical studies.

## Author contributions

X-XL: writing and drawing—original draft preparation. X-YW, Y-LZ, and J-JL: writing—review and editing. Y-LW and J-JZ: supervision. Y-LW: project administration. X-YW: funding acquisition. All authors contributed to the article and approved the submitted version.

## Funding

This work was supported by The Talent Starting Fund Project of Gannan Medical University [grant numbers QD202208 and QD202012] and The Open Project of Key Laboratory of Prevention and Treatment of Cardiovascular and Cerebrovascular Diseases, Ministry of Education [grant number XN202015].

## Conflict of interest

The authors declare that the research was conducted in the absence of any commercial or financial relationships that could be construed as a potential conflict of interest.

## Publisher’s note

All claims expressed in this article are solely those of the authors and do not necessarily represent those of their affiliated organizations, or those of the publisher, the editors and the reviewers. Any product that may be evaluated in this article, or claim that may be made by its manufacturer, is not guaranteed or endorsed by the publisher.
